# Pathogen-driven cancers from a structural perspective: Targeting host-pathogen protein-protein interactions

**DOI:** 10.3389/fonc.2023.1061595

**Published:** 2023-02-23

**Authors:** Emine Sila Ozdemir, Ruth Nussinov

**Affiliations:** ^1^ Cancer Early Detection Advanced Research Center, Knight Cancer Institute, Oregon Health & Science University, Portland, OR, United States; ^2^ Cancer Innovation Laboratory, Frederick National Laboratory for Cancer Research, National Cancer Institute at Frederick, Frederick, MD, United States; ^3^ Department of Human Molecular Genetics and Biochemistry, Sackler School of Medicine, Tel Aviv University, Tel Aviv, Israel

**Keywords:** host-pathogen interactions, machine learning, artificial intelligence, protein-protein interactions, cancer therapeutics, drug discovery

## Abstract

Host-pathogen interactions (HPIs) affect and involve multiple mechanisms in both the pathogen and the host. Pathogen interactions disrupt homeostasis in host cells, with their toxins interfering with host mechanisms, resulting in infections, diseases, and disorders, extending from AIDS and COVID-19, to cancer. Studies of the three-dimensional (3D) structures of host-pathogen complexes aim to understand how pathogens interact with their hosts. They also aim to contribute to the development of rational therapeutics, as well as preventive measures. However, structural studies are fraught with challenges toward these aims. This review describes the state-of-the-art in protein-protein interactions (PPIs) between the host and pathogens from the structural standpoint. It discusses computational aspects of predicting these PPIs, including machine learning (ML) and artificial intelligence (AI)-driven, and overviews available computational methods and their challenges. It concludes with examples of how theoretical computational approaches can result in a therapeutic agent with a potential of being used in the clinics, as well as future directions.

## 1 Introduction

Pathogens refer to organisms such as viruses, bacteria, fungi, and prion. These pathogens have evolved to adapt to their environment including multiple routes to infect a host. HPIs provide the pathogen a means to enter and dysregulate host cells (1). HPIs do not only refer to physical interactions of these two parties. It encapsulates the whole spectrum, starting from the population level to the organism level and down to the detailed molecular level, that is, physical interactions of a pathogen protein with host receptors, other proteins, and nucleic acids (1–4). This makes HPI studies important toward both a better understanding of pathogen invasion and host cell subversion strategies, as well as therapeutic development to abort these processes. One of the earliest works on HPI was by Zelle, who studied HPI in mouse typhoid caused by Salmonella typhimurium (5).

Pathogen invasion can cause pathologies in the host body, including cancer. A pathogen infection can lead to cancer through several mechanisms. Pathogen-induced senescence and chronic infection both contribute to and increase cancer susceptibility (6). Pro-inflammatory cytokines and growth factors released by senescent cells can induce proliferation in neighboring cells stimulating tumor evolution (6, 7). Pathogens frequently interfere with cellular mechanisms of host cells; they cause DNA damage and an imbalance in tumor suppressor/oncogene expression, resulting in cancer development (3, 6, 7). They can also evolve to mimic a host protein or an available PPI interface in the host. By employing this tactic, the pathogen protein can compete with a host protein for binding to the designated partner in the host metabolism. The entire host cell pathway is disrupted if the pathogen gains the upper hand in this competition (8). Human papilloma virus (HPV) is one of the pathogens that are a major risk factor for cervical cancer. They force host cells to express viral proteins with some of these, promoting the degradation of the tumor suppressor protein retinoblastoma in the host (9). Pathogen proteins expressed in host cells can also activate several host pathways that are involved in cancer (2, 7). Overall, pathogen infections are responsible for 20% of human malignancies (7), making it crucial to develop strategies to prevent pathogen infection of the human host or treatment to reverse the effects of the infection.

To develop strategy and treatment options requires understanding of pathogen entry into the host cell. Most pathogen infections begin with a physical interaction between pathogens/pathogen-released molecules and components of the host cells (1, 10). Interfering with pathogen-host physical interactions is one possible therapeutic approach for pathogen-induced cancer (11). To that end, the precise mechanism of host-pathogen PPIs (HP PPIs) is critical to comprehend. Computational studies have long guided this field (12, 13). Several databases and web servers identify PPI and HP PPI interfaces (14–20), protein-ligand interaction sites (21–23) and host protein binding pockets (24, 25). Molecular dynamics (MD) simulations (26), ML techniques (27), and AI approaches in computationally guided structural modeling, prediction of HP PPI interfaces, and drug discovery have all contributed to HP PPIs research. These data and resources assist in determining the mechanism of HP PPI and developing strategies for successfully targeting them with rationally designed small molecules and/or peptides (11).

In this review, we explain the importance of HP PPIs from a structural point of view. We establish the relationship between pathogens and cancer and how HP PPIs play a crucial role in this association. We outline known HP PPI mechanisms and discuss how different therapeutic approaches can be applied to target them. We also discuss the current landscape of computation-guided prediction of HP PPIs, including available datasets, web servers, and tools, as well as the gaps and drawbacks in these approaches. We demonstrate how predicting and better understanding of HP PPIs can help cancer research with several case studies. We conclude by discussing lessons to be learnt from these approaches and future directions.

## 2 The importance of predicting and understanding the HP PPIs

Proteins do not act alone, and more than 80% of all proteins in the cell interact with other molecules to execute their function (28, 29). The human interactome contains an estimated 650,000 PPIs with approximately 53,000 human binary protein interactions (30). They are responsible for a wide range of cellular processes such as signal transduction, transcription, replication, and membrane transport (31, 32). Protein interactions explain how proteins build metabolic and signaling pathways that allow them to perform their work (33). PPI dysregulation is frequently observed as the primary cause of a variety of pathologies, making them appealing drug targets. Proteins must have direct physical contact with their respective binding sites in order to interact, whether in a stable or transient mode (34). Such binding sites are known as “interfaces”, which are three-dimensional structures formed by groups of amino acid residues that are directly responsible for partner recognition and binding. Different PPI interfaces may have distinct structural and physicochemical properties, affinity, and binding specificity. Structural insight into the PPI interface can reveal the role of the complex in disease and its potential as a therapeutic target by providing information on its kinetics, thermodynamics, and molecular functions.

HP PPIs, like any other PPIs, require knowledge of the pathogen and host protein architectures and repertoires, their evolutionary mechanisms, and information on relevant biological data sources. Pathogens exhibit vast differences in genomic composition, evolutionary patterns, and protein function when compared to the relatively well-conserved processes found in cellular organisms. These pathogen variations are considered when studying HP PPIs (10). HP PPI interfaces can be classified as endogenous interfaces mediating intra-organism specific interactions such as pathogen–pathogen or host–host interactions, and exogenous interfaces, which mediate host-pathogen interactions.

Pathogens can interact with the host through different mediators, such as proteins, metabolites, and nucleic acids (3, 4). Pathogenic organisms or their products enter the cytoplasm of a target cell to function, survive and replicate. Pathogen proteins can interact with the host proteins by having a similarly shaped interface without sequence homology. Pathogens mimic host counterparts in sequence, motif, and interface at the structural level, permitting them to manipulate host signaling through HP PPIs (35–37). Mimicking a host PPI is a primary hijacking strategy employed by pathogens to produce a new HP PPI. These newly created interactions result in new pathways and network crosstalk. Pathogens can target essential human network hub proteins and alter cells making them acquire cancer characteristics. Therefore, creating the structural network of the ‘superorganism’ provides insight into potential pathogens transformation approaches (37). With a mimicking strategy, pathogens can alleviate host immune surveillance.

**Figure 1:**
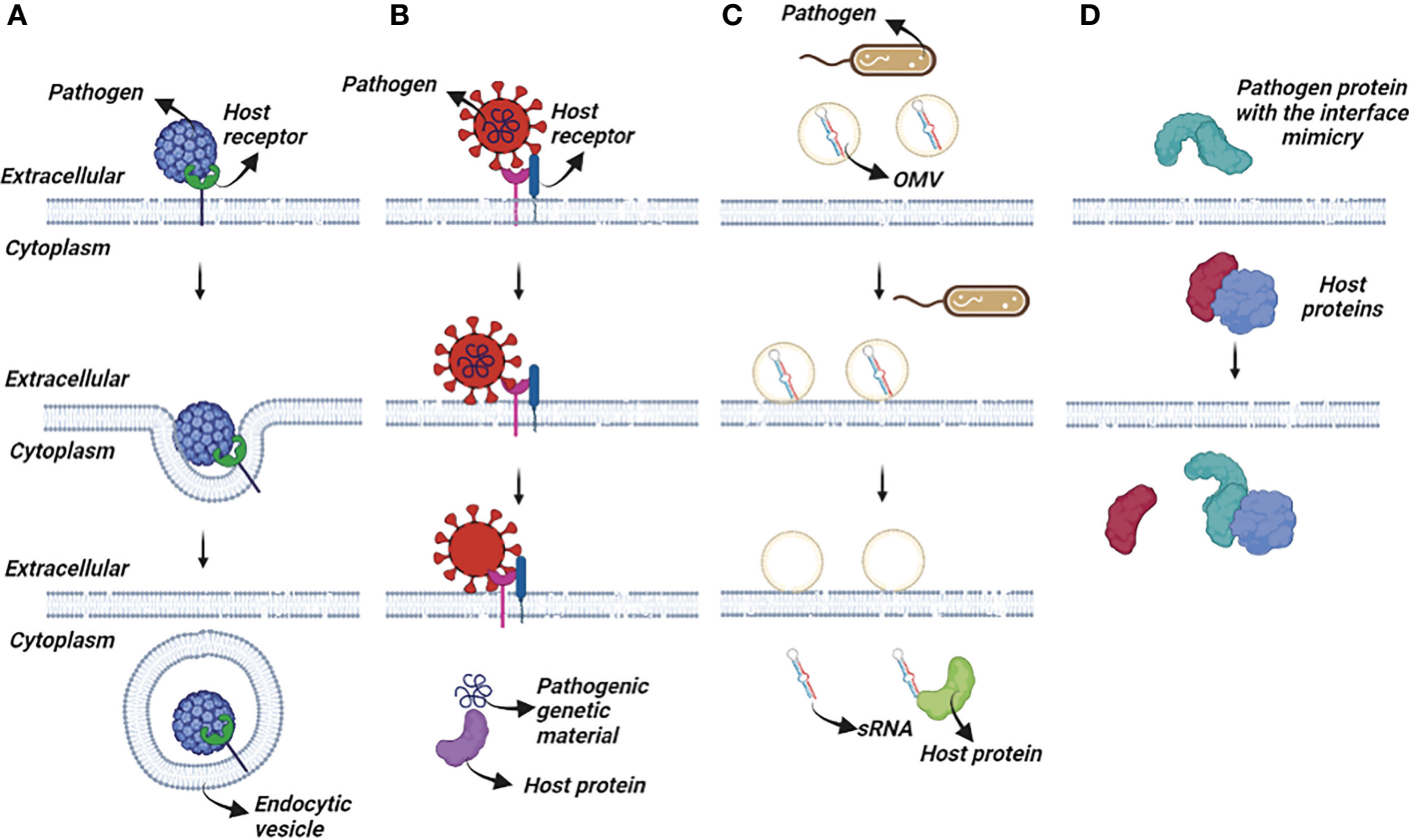
Several pathogen interaction mechanisms with the host cells. **(A)** Endocytic vesicle engulf the pathogen completely or partially after an initial interaction between host-pathogen proteins for recognition. **(B)** Pathogen proteins interact with host receptors and trigger series of cellular events. **(C)** Pathogenic OMVs release their content into the host cells. **(D)** Pathogen proteins mimic the interface of the host proteins and compete with them to bind to their partner.

**Figure 2:**
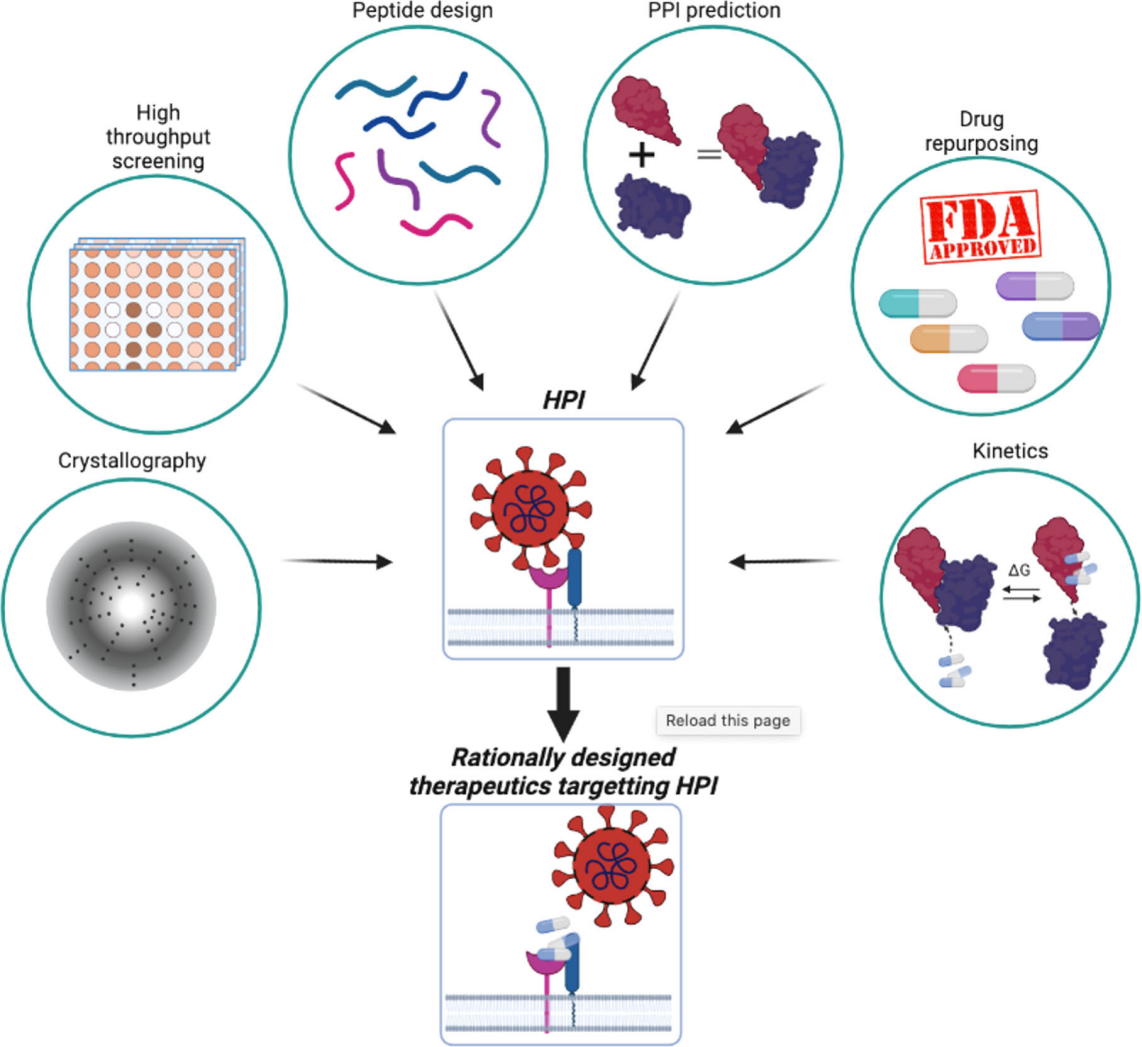
Combination of experimental and *in silico* methods aids rational therapeutics design to target HPI.

At the same time, passing through the plasma membrane is not commonly used for pathogen entry (38). Bacteria, their toxins, and parasites, never use this mode of entry. The most common solution for pathogens or their toxins is to enter the cell *via* an existing entry mechanism established by exogenous interactions or for small metabolites, diffusion. These entry mechanisms can be studied using three different approaches (**Figure 1**). Ligand-induced endocytosis can occur in the cellular membrane, allowing pathogens to enter the host cell *via* endocytic vesicles (39). The SV40 virus (40) is an example of ligand-induced endocytosis (**Figure 1A**). Extracellular interaction between a pathogen protein and a host receptor cripples cellular events, including dysregulation of gene expression, enhancing/preventing specific signaling cascades, and even allowing pathogen genomic material to enter the host cells (**Figure 1B**). Interaction of the viral spike protein of SARS-CoV-2 and angiotensin-converting enzyme 2 receptor of the host allows viral genomic material to enter host cells (41). The initial attachment of the SARS-CoV-2 virus to the host cell is initiated by this extracellular interaction. Finally, bacterial outer membrane vesicle (OMV)-mediated protein delivery to host cells is an important HP PPI mechanism (**Figure 1C**). According to new evidence, OMVs contain differentially packaged short RNAs that have the potential to target host mRNA function, such as stability. Gram-negative bacteria, such as P. aeruginosa, produce OMVs, which are important for host colonization (42). The content of OMV, the endocytic vesicle genetic material released into the host cell further interacts with host proteins to interfere with normal cellular functions and induce the expression of their own proteins. Additionally, as noted above, mimicking host protein interfaces give pathogen proteins an advantage in interacting with host proteins (**Figure 1D**). A better understanding of HP PPIs is critical for the development of new therapies, such as small molecules capable of binding and blocking pathogenic interactions (**Figure 2**) (43, 44). Current drug discovery process has three major steps: identifying a potential drug target, studying its properties, and designing a corresponding ligand (45). Insight into protein-protein interactions can help in the design of molecules that target protein complexes involved in these pathologies (**Figure 2**). The action of Maraviroc as an inhibitor of HIV-1 entry into host cells is a classic example of HPIs being blocked at the interface level. This drug binds to the cellular co-receptor CCR5, preventing it from interacting with viral GP120 protein, which is required for HIV-1 infection (46).

However, studying HP PPIs and developing drugs to target them are challenging tasks. A variety of factors can exacerbate the difficulty of identifying small molecules that inhibit such interactions. Some of the challenges include the flatness of the interface, the lack of a small molecule backbone as a starting point for drug design, the difficulty of characterization of the binding kinetics, and the size of small molecule libraries (47). Advances in molecular biology, chemistry, and computational modeling techniques have made progress in alleviating some of these issues, with some approaches creating small molecules that target HP PPIs (**Figure 2**). FDA-approved drugs are pre-screened and tested experimentally (48). Screening aids in identification of drugs that bind to interfaces and compete with the native binding partners (11, 49). Interacting partners can be identified *in silico*, making these approaches widely used in drug design, including repurposing applications (**Figure 2**) (50). The druggability of protein-protein interactions with small molecules have been challenging. However, with advancements, including in computational modeling and experimental structural methods such as X-ray crystallography, NMR and cryo-EM, and innovative strategies, involving e.g., bifunctional small molecule ligands, as in the case of K-Ras (51), fragment-based drug discovery (52), and orthosteric and allosteric (53) PROTACs, there have been some successes. Several studies on the kinetics and thermodynamic properties of protein-protein interactions have also greatly contributed to a better understanding of their affinity (54). Currently, PPIs can be considered as challenging, but druggable.

The available PPI data was estimated to represent only a small percentage of all PPIs in humans (55), and it may not be a good representation of the entire interactome. As relatively little is known, *in silico* approaches are emerging to help fill in the gaps. Before discussing how HP PPI can aid in the treatment of pathogen-driven diseases such as cancer, the following section describes the current state of the art in *in silico* guided PPI prediction approaches that can be used for HP PPI prediction and targeting.

## 3 The current landscape and gaps in computer guided prediction of HP PPIs

Predicting PPI involves predicting proteins that interact as well as decoding their binding interfaces and the structures of their complexes. The limitations of experimental methods make computational prediction of PPIs an essential strategy. Structures and sequences are required for computer-aided PPI prediction. Docking/simulation-based and data-driven/ML-based approaches are adapted to use these data for prediction (56, 57).

The interaction interfaces and complex structures can be predicted using docking/simulation-based techniques. Rigid body or flexible docking can be used to evaluate surface complementarity. Flexible docking involves a significantly larger number of coordinates, whereas rigid body ignores the conformational changes between the bound and unbound states (57). Simulation-based methods can obtain a more refined structural model of the complex. MD simulations can compute the interaction strength and the conformational changes upon PPI formation using a force field to represent atomic interactions and capture the entropic contribution in the ensembles (58). These techniques often employ physics-based and/or geometric models to look for probable conformations with low interaction energy and high surface complementarity (57). Through the sampling of the free energy landscape, simulation-based methods capture kinetics, mechanisms of action and binding affinities. Because simulation timescale of binding events involve high computational cost, techniques that include MD simulations and docking are typically used to explore the dynamics of interactions or to evaluate their strength rather than to identify which proteins interact with one another and form a PPI network (54). Although binding events normally require reaching at least the millisecond timescales, current simulations frequently sample nanosecond to microsecond timescales (59).

One of the most significant differences between docking/simulation-based methods and data-driven methods is that the latter have a capacity to be used on a large scale. As many proteins still lack structural information, the data-driven/ML approaches that use sequence data are also frequently applied. Although new methods are being developed employing structure, most of the data-driven/ML approaches still use sequence-based information (60, 61). These methods can be used to extract features such as evolutionary information, particularly correlated mutation analysis, and secondary structures from the protein sequences. On the other hand, structure-based data-driven/ML methods use conserved interfaces among homologs as templates to study and identify the interface of PPI of interest (62, 63). ML-based methods require input data obtained from datasets of experimentally determined or other available interfaces to train their algorithms. The trained algorithms are used to predict the interface of a PPI of interest (64, 65). Co-evolution based statistical models can be considered as data driven as they utilize multiple sequence alignment data. Interface residues are likely to coevolve, and the alignment data is used to identify such residues (66). Recent biotechnological advances are generating a wealth of protein data, helping data-driven computational approaches improve their performance.

Computer-guided PPI prediction approaches can also use metabolic pathway mapping, gene neighbor, and domain fusion analyses. Research suggests that proteins involved in a coupled enzymatic reaction can form a temporary complex (67). A gene neighborhood method has explored the functional linkages between two proteins. The assumption here is that if two proteins are found in the same neighborhood in different genomes from different organisms, they are highly likely to be functionally linked (68). Especially noteworthy is the emergence of AI-driven methods (69, 70), which are already being optimized and applied to prediction of PPI (61, 71). The landmark AlphaFold does not involve a homology-based strategy, nor does it model the prediction on available PDB structures, as typical homology-based methods do. Instead, it uses the PDB structural database to learn structural patterns (69, 71, 72). We expect AI-driven approaches to dominate the structural modeling of the HP PPIs field in the future. Still, AI-driven methods, such as AlphaFold, are not perfect. They are challenged by dynamic energy landscapes of biomolecular function and allosteric mechanisms (73). To associate structure and function, the populations and relative energies in protein ensembles should be considered. AlphaFold predictions are unable to directly address it despite its impact. This functional goal can only be achieved by their sampling (71).

**Figure 3:**
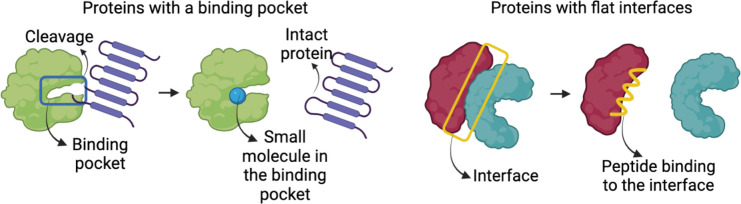
Representation of a protein with binding pockets/cavities and proteins with large flat interfaces.

Another challenge for predicting PPI is related to the geometric features of the interfaces. Protein structures and their interaction interfaces are not uniformly shaped. Most enzymes have binding pockets shaped as cavities (**Figure 3**, left), whereas many PPI interfaces have large flat surfaces (**Figure 3**, right). This makes interface prediction and the drug design process difficult, because determining where the drugs would bind is the first crucial step (47). To address these issues and predict a flat binding interface, some approaches are being developed (74, 75). Identifying residues with higher contribution to the binding affinity and protein recognition is one of them. Most of the affinity is provided by a small set of residues at interfaces known as hot spots (76). Hot spots are the primary targets of small compounds designed to disrupt PPIs (50, 77). Below are databases and prediction methods for find flat binding interfaces and cavities. They include structural information, kinetics, thermodynamics, and molecular functions of the PPI and their interfaces. **Table 1** summarizes some representative online protein binding pocket prediction tools as well as interface and PPI databases that are available. There are many databases with a large spectrum of approaches that can be used to study HP PPIs. DeepSite, for instance, predicts putative ligand binding sites in proteins using deep convolutional neural networks (23). Protein structures are treated as 3D images, and hydrophobic and aromatic features, hydrogen bond acceptor or donor, charges, and metallic properties are assigned to each pixel based on the atoms in it. Each pixel is assigned as positive or negative based on the distance of each pixel to the geometric center of the pocket. The threshold for negative and positive assignments was selected with consideration of common binding sites from the literature. Their neural networks use these data from the literature to train its algorithm to predict a binding site.

**Table 1:** List of available representative methods/algorithms/databases for protein binding pocket prediction, PPIs and PPI interfaces.

Name	Website link
Protein binding pocket prediction methods	
DeepSite	https://www.playmolecule.com/deepsite/ (24)
PockDrug	http://pockdrug.rpbs.univ-paris-diderot.fr/ (25)
AlloPred	http://www.sbg.bio.ic.ac.uk/allopred/home (78)
POCASA	http://altair.sci.hokudai.ac.jp/g6/service/pocasa (79)
PPI and PPI interface databases	
PIFACE	http://prism.ccbb.ku.edu.tr/piface (14)
PLIC	http://proline.biochem.iisc.ernet.in/PLIC/index.php (21)
ProtCID	http://dunbrack2.fccc.edu/ProtCiD/default.aspx (80)
3did	https://3did.irbbarcelona.org (81)
sc-PDB	http://bioinfo-pharma.u-strasbg.fr/scPDB (22)
HPIDB 3.0	https://hpidb.igbb.msstate.edu/ (16, 17)
MINT	https://mint.bio.uniroma2.it/mint/ (19)
DIP	http://dip.doe-mbi.ucla.edu (15)
IntAct	http://www.ebi.ac.uk/intact (82)
BIND	http://www.bind.ca/ (83)
BRITE	http://www.genome.jp/brite/ (84)
BID	http://tsailab.org/BID/ (85)
STRING	https://string-db.org/ (86)
Viruses. STRING	http://viruses.string-db.org/ (87)
HHPID	https://www.ncbi.nlm.nih.gov/genome/viruses/retroviruses/hiv-1/interactions/ (88)
InterSPPI	http://zzdlab.com/InterSPPI/ (18)
HMI-PRED 2.0	https://hmipred.org (20, 89)
BioGRID	https://thebiogrid.org/ (90)
HPRD	http://www.hprd.org/ (91)
HCVPro	http://cbrc.kaust.edu.sa/hcvpro/ (92)
VirHostNet	https://virhostnet.prabi.fr/ (93)

AlloPred makes predictions of allosteric pockets on proteins by perturbing normal modes (78). AlloPred simulates how the dynamics of a protein would change if a modulator were to occupy a particular pocket. They used the Fpocket algorithm (94) to initially locate the pockets on the protein. The elastic network model was then used to determine the normal modes. At the active site, the impact of this disruption (binding) was measured. The findings were integrated with output from Fpocket in a support vector machine (SVM). DeepSite and AlloPred, are two different approaches showing that both simulation-based and data-driven approaches can be adapted for similar purposes.

The variability in PPI interface datasets comes from both the protein types that are listed in such databases and the methods used to create them. PIFACE present all available PPI clusters (until 2012) *via* their interface structures. They identified 22,604 unique interface clusters. These clusters can be used to identify and investigate both shared and unique protein binding sites (14). HPID3.0 is a curated database tailored for HP PPIs (17). There are still more specialized databases available, such the HCVpro, PPI database for the hepatitis C virus (HCV) (92). The HCVpro is a knowledgebase database that contains consolidated information on PPIs, functional genomics, and molecular data that was gathered from various virus databases (95–97). VirHostNet combines one of the largest human interactomes (10,672 proteins and 68,252 non-redundant interactions) reconstructed from publicly available data with an extensive and unique dataset of virus-virus and virus-host interactions (2671 non-redundant interactions) representing more than 180 different viral species (93). Noticeably, the methods for predicting PPIs and their interfaces have clearly improved. Nonetheless, for computer-aided PPI predictions (CAPP), like any other prediction methods, several challenges are still relevant (32, 93). Some of the challenges and gaps are common to all methods mentioned above, while others are specific to the prediction approach environment.

Some of those common challenges for PPI prediction are related to the nature of PPIs. Transient (domain–motif) interactions are not well-covered in available PPI databases. Stable PPIs typically use large interfaces, whereas transient PPIs use short linear peptides making the prediction more difficult (10). Also, because of the lack of conservation across species, conservation-based analysis is inapplicable to transient interactions (32). Not only the transient interactions but the dynamic nature of proteins is one of the most prominent challenges for CAPP (98). Including protein flexibility in the calculations is computationally highly expensive. Therefore, many CAPP tools consider protein structures as rigid bodies (33). The distinction between protein isoforms is another critical issue. PPI of only one protein isoform is frequently listed in the databases, though it is unknown whether any other isoform interacts with the same partner *via* the same interface (99). Furthermore, CAPP algorithms determining which proteins interact with which may predict multiple complexes as feasible from energy or/and structural point of view. However, in reality, it may not be feasible for them to be in physical contact, since they do not share the same temporal and spatial compartment (100).

Advanced computational techniques such as ML and deep learning (DL) suffer from different types of drawbacks. A large amount of data is needed for model training. A model might not be able to learn the rules because only a few thousand structures of complexes are available across the databases. Data imbalance is a challenge in any learning process. Third, there is a requirement for high-quality experimental data because a model cannot afford to learn from low-quality data (101).

HP PPI-focused algorithms and databases have their own set of challenges. There is still a lack of domain knowledge for some viruses with genomes larger than 20 kb, and many of their proteins have neither an assigned domain nor a known function (10). The fact that HP PPI prediction is based on a diverse range of experimental methods, as well as having curation error and redundancy, are additional problems (102). Moreover, certain databases provide pathogen data at the species level disregarding interspecies interaction (17, 87).

Overall, CAPPs need rigorous validation because they are error prone. Although there are many protein-protein docking programs, there are few ways to systematically assess the predicted PPI complexes (64). International benchmarking studies, such as critical assessment of predicted interactions (CAPRI), show how imperfect predictive methods are and how they can be improved. Effectiveness depends on the capacity to curate and derive the best knowledge input. Along these lines, additional structural information about protein complexes will significantly increase the performance of computational methods, enhancing their capability (103). Notwithstanding, CAPP approaches are already helping in predicting, and thereby clarifying how HP PPIs can aid in studies of cancer. In the next section we discuss a few case studies that demonstrate the importance of CAPP approaches in HP PPI and cancer research.

## 4 How predicting HP PPIs can help cancer research

The precise molecular mechanisms by which pathogens rewire the host pathways to trigger malignant transformation remain unknown, despite the abundance of data on their role in cancer. Pathogens can have an impact at any stage of the multi-step process of carcinogenesis. Here, we discuss some examples from the literature documenting how insight into HP PPI mechanisms can help cancer and pathogen-driven cancer research.

Frisan (104) explains that interaction of bacterial toxins with subcellular membrane compartments induce DNA damage and trigger the DNA damage response (DDR). Normally, damaged cells halt the cell cycle and, by activating DNA repair mechanisms, induce senescence or apoptosis (105, 106). However, cells with toxin-induced DNA damage are more likely to survive and bypass the DDR-induced cell death or cellular senescence, resulting in genomic instability. Chmiela et al. (107) and Gagnaire et al. (7) discuss possible mechanisms of gastric cancer initiation in response to H. pylori infection. Similarly, DNA damage combined with impaired repair processes, as well as mitochondrial DNA mutations, make infected cells more susceptible to tumor growth (108). Although the expression of DNA mismatch repair (MMR) proteins increases in response to DNA damage, H. pylori-induced gastric inflammation impairs MMR (109). Some of the bacterial proteins activate the PI3K-AKT-MDM2 pathway, which causes p53 degradation in gastric epithelial cells (7). Gagnaire et al. also describe how different host pathways that are activated in cancer are also activated by bacteria as part of their infection cycle. These include the nuclear factor-κB (NF-κB), PI3K-AKT, MAPK, and β-catenin pathways. In addition to causing DNA damage, bacteria can disrupt the DDR by attacking important participants in the response, such as p53. Oncoviruses known to target p53 to promote cellular transformation include the HPV, hepatitis B, hepatitis C, and Epstein-Barr virus (EBV), some bacteria may also do this.

**Figure 4:**
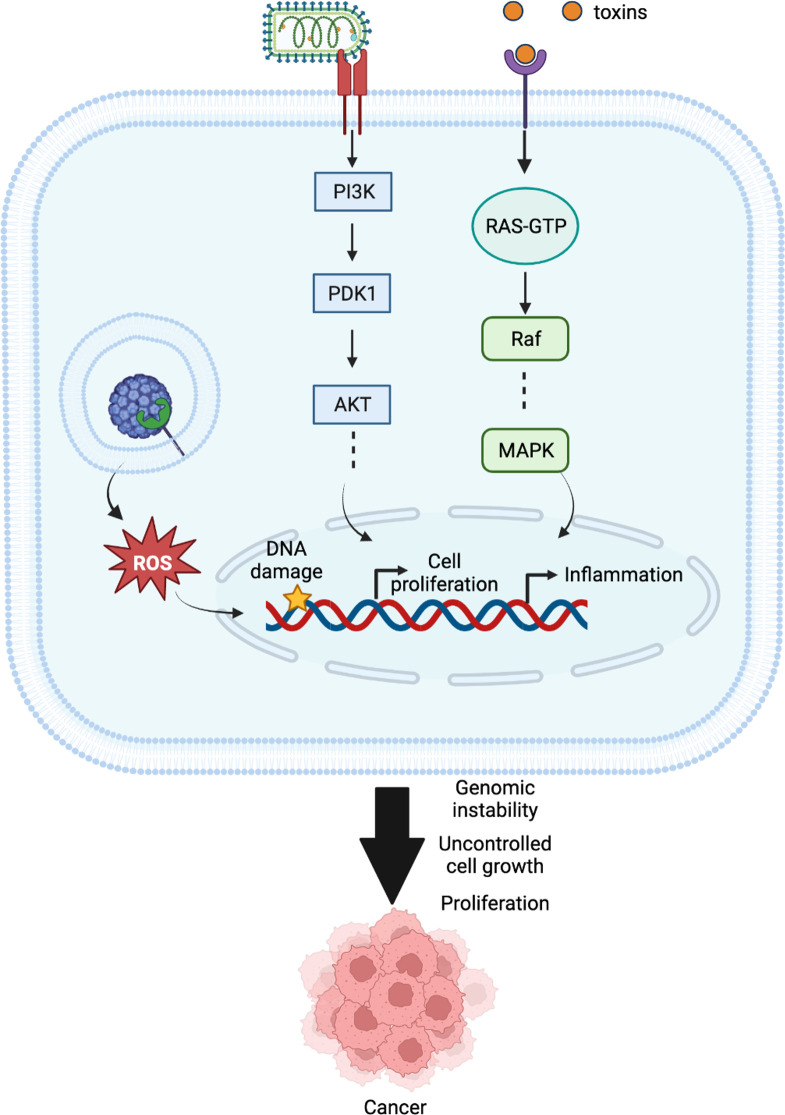
Different cellular mechanisms interfered by pathogens in host cells that might result in cancer.

In brief, the first impacts of a pathogen on a host cell are the production of reactive oxygen species, DNA damage, imbalances in the activity of cellular pathways including but not limited to MAPK and β-catenin activation, and p53 degradation. These responses are followed by an inflammatory state, immunosuppression, and the impairment of DNA damage repair mechanisms. These lead to genomic instability, uncontrolled cell growth, and proliferation, which are some of the hallmarks of cancer. **Figure 4** describes how HPI mechanisms can cause these responses and lead to emergence of cancer, and how advanced computational methods can help decipher these mechanisms. By studying related HPI mechanisms, it is possible to find new drug targets, biomarkers, and therapeutic candidates for cancer treatment.

People living with HIV are more likely to develop the following cancers: Kaposi’s Sarcoma, non- Hodgkin’s lymphoma, and cervical cancer (in women), and as the HIV-positive population ages, the incidences of colon, breast, and prostate cancer also increase (110). Further, since people with HIV are more vulnerable to chronic infections, they can develop HPV-associated tumors. Cervical and anal cancers are the most common HPV-related tumors in HIV patients (111). HPV is thought to be responsible for 30% of the head and neck cancers and the majority of oropharyngeal cancers (112). Hence, generating a HIV-human interactome becomes important (88, 113). Brass et al. identified a set of proteins important for HIV survival (HIV-dependency factors (HDFs)) (113). Pinney et al. compared this list of HDFs with the HHPID database of curated HIV interactions and identified 36 overlapping proteins (88). Then, based on the gene ontology terms associated with the HDFs, they discovered that the HDFs are associated with cellular processes such as mRNA transport, protein transport, and lipoprotein biosynthesis. Thus, stopping the spread of AIDS will aid in the fight against cancer, providing information on HIV-host interactions, which along with the potential of a systems biology approach, will be invaluable in drug development.

The Epstein-Barr virus is an oncovirus that secretes the lytic-cycle protein BARF1 to undermine host immunity by interacting with human cytokine CSF1 (114). Guven-Maiorov et al. used HMI-PRED to successfully pinpoint the BARF1-CSF1 complex and the interaction surface (8, 89). Then, in addition to discovering the interaction of the BARF1-CSF1 complex, they presented 155 new potential HP PPIs for the BARF1 protein. Some host immunity proteins, including the T-cell receptor beta 1 chain C region (TRBC1), immunoglobulin constant heavy chains (IGHε), and tumor necrosis factor (TNFα), are among the 155 potential targets. These findings imply that the BARF1 protein can influence alternative host immunity pathways in addition to the canonical pathway (CSF1R). These potential HP PPIs and the structures of their complexes could offer a mechanistic understanding into how EBV evades host recognition and survives for years. They also built an integrated structural network with structures for all pairwise interactions for oncoviruses and their human hosts (8). This network helps to identify some hub proteins, including UBC, UBB, B2MG, A102, CALM2, and TRBC1, that are commonly targeted by oncoviruses. As interface mimicry is a more common strategy for the pathogens than mimicking the whole protein structure, interface-based methods are more successful than global structure similarity-based methods to identify the HP PPIs. Large-scale application of interface-based methods has the potential to improve the HP PPI predictions (36). Dyer et al. used experimentally discovered interactions between human proteins and proteins from B. anthracis, F. tularensis, and Y. pestis to create an HPI network for each (115). They conducted a network analysis using the GrapHopper algorithm (116), defined the Conserved Protein Interaction Module (CPIM), and discovered that pathogen proteins have a propensity to interact with human proteins that act as hubs and bottlenecks in the human PPI network. They noticed that the three networks contain hubs of conserved human proteins like NF-κB. Additionally, they discovered that some Y. pestis proteins can interact with CXC-chemokine receptor 4 (CXCR4), which is a promising new target for anti-HIV medications because of its role as a main coreceptor for the human immunodeficiency virus (117). These novel networks aid in the discovery of new interactions that are important in pathogenesis and host response and can be applied toward the discovery of vaccines and immunotherapeutics.

Lin et al. (118) adapted a drug repurposing strategy powered by ML models to address the urgent need for an effective anti-HPV drug. They built, tested, and chose machine learning predictive models to predict antivirals that might potentially interact with HPV proteins. To perform the comparatively extensive *in silico* screening for the 9 HPV-16 protein, they gathered and examined 96 FDA-approved antiviral drugs. They were able to correctly predict 57 pairs of antiviral-HPV protein interactions out of 864 pairs of antiviral-HPV protein associations made up of the combination of 9 HPV-16 proteins and 96 antiviral drugs. One of the drugs they identified as a potential anti-HPV treatment is docosanol, which was predicted to interact with HPV-16 protein E7. Docosanol is an FDA-approved anti-EBV drug. Interestingly, a recent clinical case report claimed that HPV infection were successfully treated using Docosanol, curcumin, and other medications in combination (119). For the development of anti-HPV drugs, Lin et al. produced promising drug candidates.

**Figure 5:**
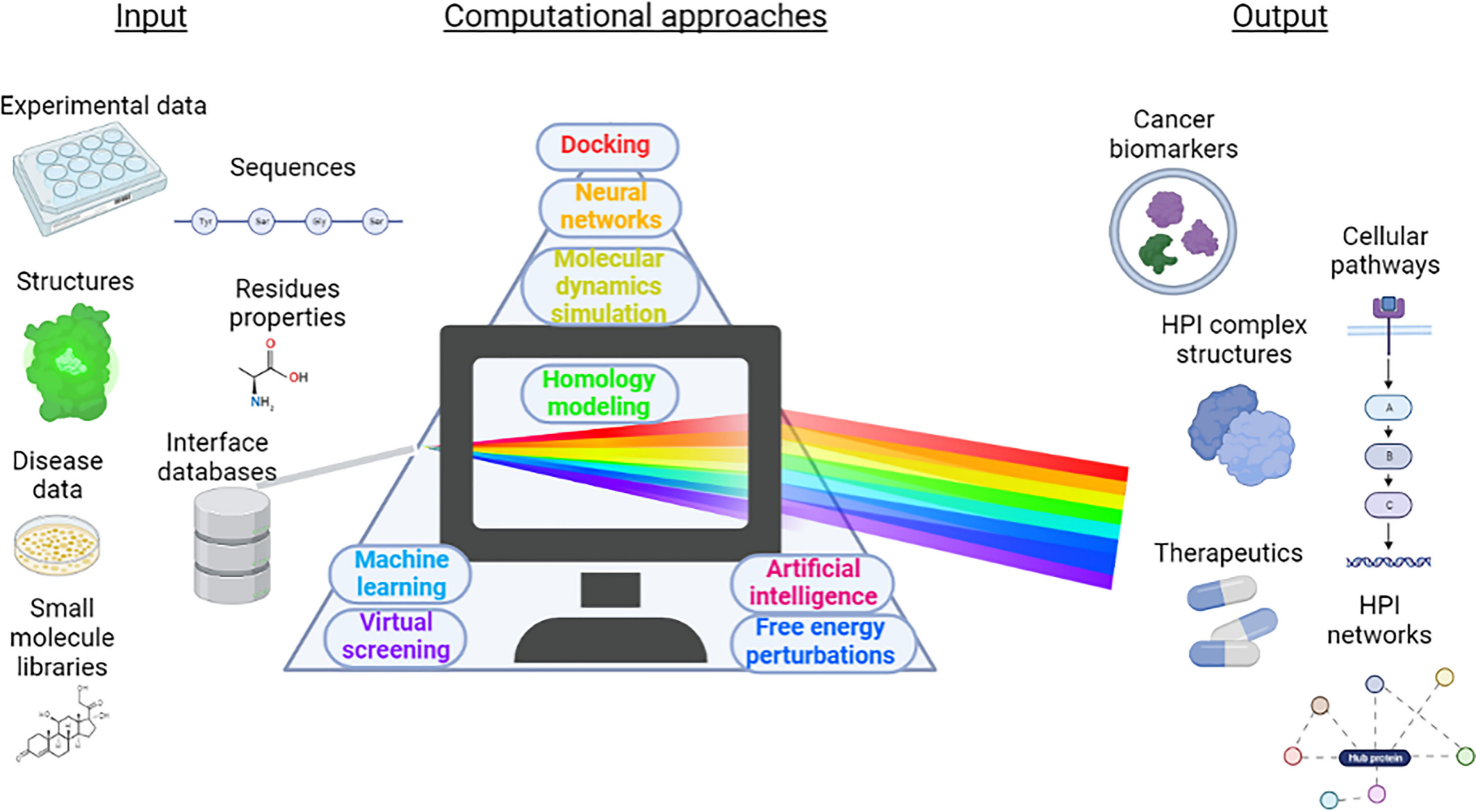
A scheme of benefits that can be obtained from computational approaches in HPI-related cancer research. Data such as experimental analysis, available protein structures, available PPI interface databases, amino acid residue properties, small molecule libraries, disease data and amino acid sequences of the proteins of interest provide input. Various computational methods including but not limited to MD simulations, docking, ML, AI, neural networks, free energy perturbations, virtual screening process these data and can identify novel cancer biomarkers, unravel unknown HPI complex structures, produce HPI networks, screen for novel therapeutics and identify cellular pathways that they impact.

These studies, which have been compiled from a variety of sources, demonstrate the wide range of computational approaches to identify new HPIs and cellular mechanisms that are affected by pathogen infections. They can help in screening novel or repurposed drug compounds against HPI complexes, and in producing drug candidates. A framework that summarizes the benefits of these studies is shown in **Figure 5**. Input data from different sources including amino acid residue properties, protein sequences, experimental data, protein 3D structures, available PPI interface databases, as well as tissue locations, can be processed using different computational techniques and databases discussed throughout the review. Proper operation of these approaches can help to discover pathogen-driven cancer biomarkers, host-pathogen complex structures, pathogen induced cellular pathways, therapeutics against the pathogens and HPI networks.

## 5 Conclusions and future perspectives

Since PPIs are crucial for cell function, studies of protein interactions are essential to comprehend how biological systems operate. Aberrant, pathologic HPIs can influence downstream target genes, resulting in a variety of diseases, including cancer.

HPI interface identification with high accuracy has many applications in computer-aided rational drug design. Although computational prediction of protein interfaces has made significant strides in recent years, there is still much room for improvement and innovation. The bottlenecks and drawbacks can be divided into two main categories: shortcomings of computational approaches and challenges due to the nature and physicochemical characteristics of the HPI. Traditional protein docking algorithms can work for complexes consisting of two partners. Increasing the number of proteins in the complex challenges these methods. In protein-protein docking, deeper pockets on the protein surface as well as the presence of hot spot residues, diversify the HPI and may, or may not, assist in accurate prediction (56). An intrinsic bottleneck in docking, especially of proteins and small ligand inhibitors, is protein flexibility, which is challenging to include, and algorithms which produce rigid models, like AlphaFold, cannot (71). Recent applications also emphasize the need for improvements in the scoring functions, which pose a further challenge. ML and DL techniques can help and are increasingly incorporated in prediction algorithms. However, they depend on the data that they learn, which may, or may not, have abundant candidates in the ‘correct’ conformation. For example, consider that drug targets are frequently the ‘active’ conformation, however, the PDB populates the more stable inactive structures. That is, the biologically relevant state of the protein may not be sufficiently populated in the docking ensemble. High throughput data can offer useful co-evolutionary information. Inverse-covariance-matrices have recently made significant improvement in protein structure prediction (120). However, data is mixed as far as their usefulness in predictions of protein complexes. Consider that pathogens are unlikely to possess the time for their evolutionary acquisitions. These hurdles hamper the predictions and design of potent molecules that can destabilize (or, if repressor, stabilize) HPIs. While there is progress and there are successes, further improvements will have a significant impact on HPIs in disease pathways (101). Together with the vast amount of protein sequences currently available, the PDB has been building up a large number of atomic resolution structures, which provide learning and training data for machine learning algorithms (56, 121) and functional information.

In summary, even though it is still not possible to predict HPI interactions with ‘perfect’ accuracy, computational methods can predict the most likely interaction models based on the input data. These interactions can act as the basis for further experimental studies. When combined, information on gene expression and protein interactions are expected to increase the confidence in the HPI and the corresponding HPI network. Recent advancements are also paving the way for the generation of networks for identifying HPI and signal transduction pathways playing role in cancer, all toward rational drug design.

## Author contributions

EO: Conceptualization, writing-original draft, writing-review and editing. RN: Supervision, conceptualization, writing-original draft, writing-review and editing. All authors contributed to the article and approved the submitted version.
